# Minimally Invasive Open Reduction and Maintenance Technique for Anterior Sacrococcygeal Dislocation: A Case Report

**DOI:** 10.3390/medicina59111958

**Published:** 2023-11-06

**Authors:** Chang-Hyun Kim, Sung-Joon Yoon, Soon-Do Wang, Woo-Jong Kim, Chang-Hwa Hong

**Affiliations:** Department of Orthopedic Surgery, Soonchunhyang University Cheonan Hospital, 31, Suncheonhyang 6-gil, Dongnam-gu, Cheonan 31151, Republic of Korea; osdrchkim@gmail.com (C.-H.K.); yunsj0103@naver.com (S.-J.Y.); 118539@schmc.ac.kr (S.-D.W.); kwj9383@hanmail.net (W.-J.K.)

**Keywords:** sacrococcygeal region, joint dislocation, open reduction, minimal invasive surgery

## Abstract

Anterior dislocation of the coccyx is rare, but it can occur due to trauma. Conservative treatment is usually performed. However, dislocation reduction may be required to control severe pain in the acute phase or to prevent chronic complications. If manual reduction fails, open reduction is required. The extent of the incision and the method used to maintain the reduction should be considered during open reduction. A 56-year-old male patient experienced a dislocation of the sacrococcygeal joint after falling backwards. Despite conservative treatment, the patient complained of persistent pain during sitting and when using the bathroom. A manual reduction was attempted but failed. We performed joystick reduction via minimal incision and maintained the reduction using a one-strand trans-osseous suture passing through the skin. The patient was advised to use a soft cushion when sitting or lying down for four weeks after surgery. The supine position was not restricted. The patient’s symptoms significantly improved after surgery. At the 6-month follow-up, the sacrococcygeal joint showed good alignment and no surgical complications occurred. During the treatment of sacrococcygeal dislocation, the rapid alleviation of acute pain and minimizing potential complications are key points. If open reduction is needed, the minimally invasive reduction technique with a one-strand trans-osseous suture may offer patient satisfaction and a good surgical outcome.

## 1. Introduction

Sacrococcygeal joint dislocation is a rare condition that can occur as a result of trauma, particularly falling backwards. A dislocation of the coccyx can cause strain or damage to the surrounding tissues, leading to inflammation and pain. It can cause significant discomfort and interfere with daily activities. In most cases, conservative treatment is used to control the associated pain by reducing direct pressure and stimulation. This includes physical therapy, the injection of steroids into the joint, taking oral medications such as analgesics and muscle relaxants, as well as using cushions. However, in some cases, surgical treatment, including coccygectomy, may be required [1,2]. To our knowledge, few studies have reported the treatment of sacrococcygeal joint dislocation. No standardized guidelines are available for dislocation reduction. However, malalignment due to dislocation itself can potentially interfere with activities such as sitting, defecation, sexual intercourse, or childbirth in women [3]. Additionally, a residual instability of the dislocated joint can lead to delayed post-traumatic coccygodynia [4]. To prevent such complications, dislocation reduction can be attempted in the acute phase, and if closed reduction fails, open reduction may be warranted. In open reduction, factors associated with the extent of the incision and the method for maintaining reduction may be considered. We present a case of successful treatment of an anteriorly dislocated coccyx that was not reducible manually, highlighting the surgical technique, including mini-open joystick reduction and a relatively easy procedure for maintenance.

## 2. Case Presentation

**Figure 1:**
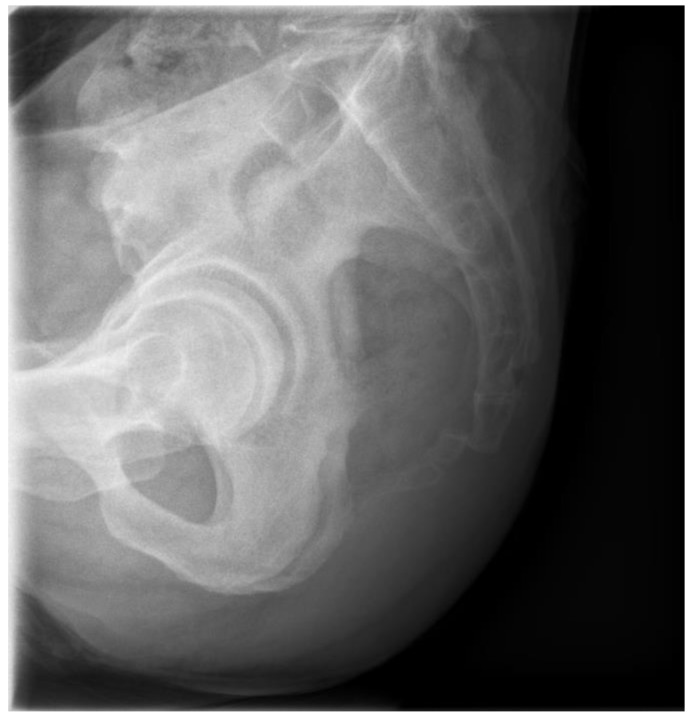
Initial plain lateral radiography shows anterior dislocation of the coccyx.

A 56-year-old male patient presented to the outpatient clinic three days after experiencing coccygeal pain due to a backwards fall. He complained of severe tenderness in the coccygeal area, but no bruising or external wounds were observed. No significant signs of neurological injury were detected. The patient had no underlying disease except hypertension. On plain radiography, the coccyx was anteriorly dislocated from the sacrococcygeal joint (Figure 1).

The patient was educated about conservative management. He was instructed to use a soft cushion when sitting, to avoid pressing the coccyx as much as possible, and to take laxatives if he had difficulty in defecation. Analgesics and muscle relaxants were prescribed. One month later, the patient returned to the clinic with persistent pain, particularly when sitting on a chair and using a bathroom. A week before the follow-up visit, the patient visited a nearby hospital due to pain. A closed reduction through the rectum was attempted, but the pain was not alleviated. The follow-up plain radiography still showed anterior dislocation of the coccyx. Surgical reduction was planned considering the possibility of chronic coccygodynia due to instability and malalignment.

**Figure 2:**
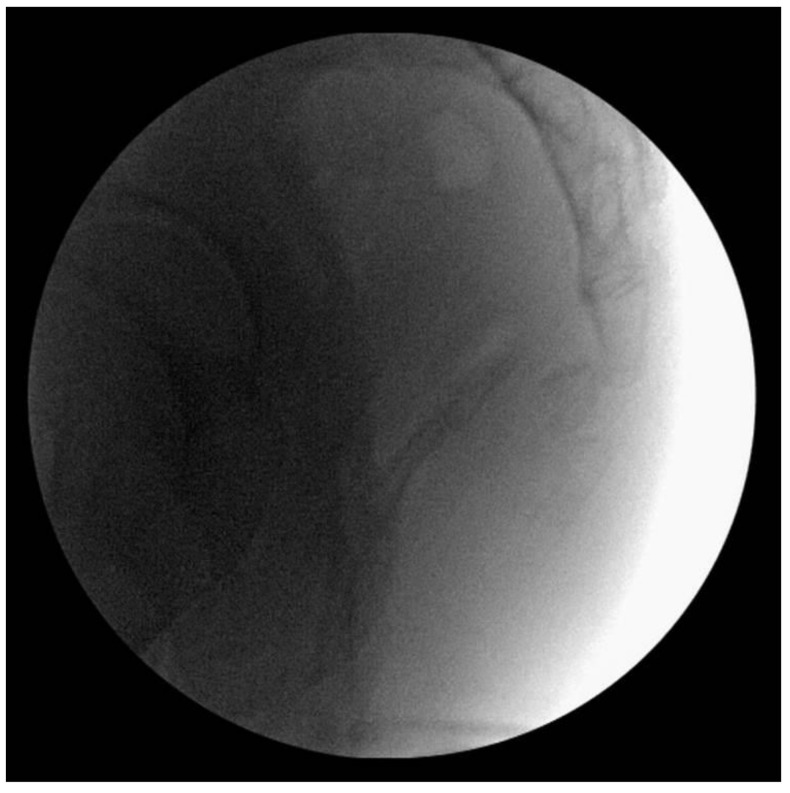
Intrarectal manipulation was attempted before open reduction, but the dislocated coccyx was not reduced.

The patient underwent surgery under general anesthesia in a prone position. Before making an incision, manual reduction was attempted via the rectum under intraoperative fluoroscopy, but it failed (Figure 2).

**Figure 3:**
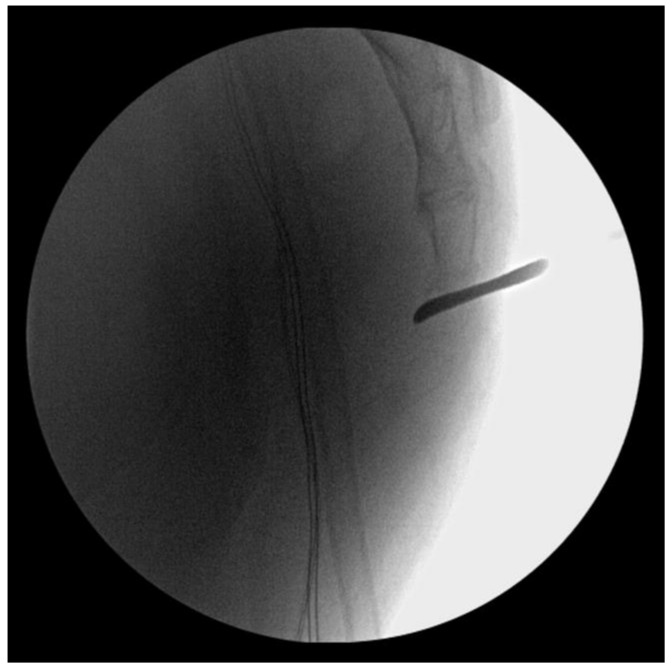
Joystick reduction of the dislocated coccyx using a freer periosteal elevator like a lever.

To avoid additional injury to the rectal mucosa, we decided to perform an open reduction. A stab incision was made in the posterior aspect of the sacrococcygeal joint. A freer periosteal elevator was inserted through the incision, reaching the anterior periosteum of the joint; it was placed like a lever on the distal sacrum to hold back the coccyx posteriorly (Figure 3).

**Figure 4:**
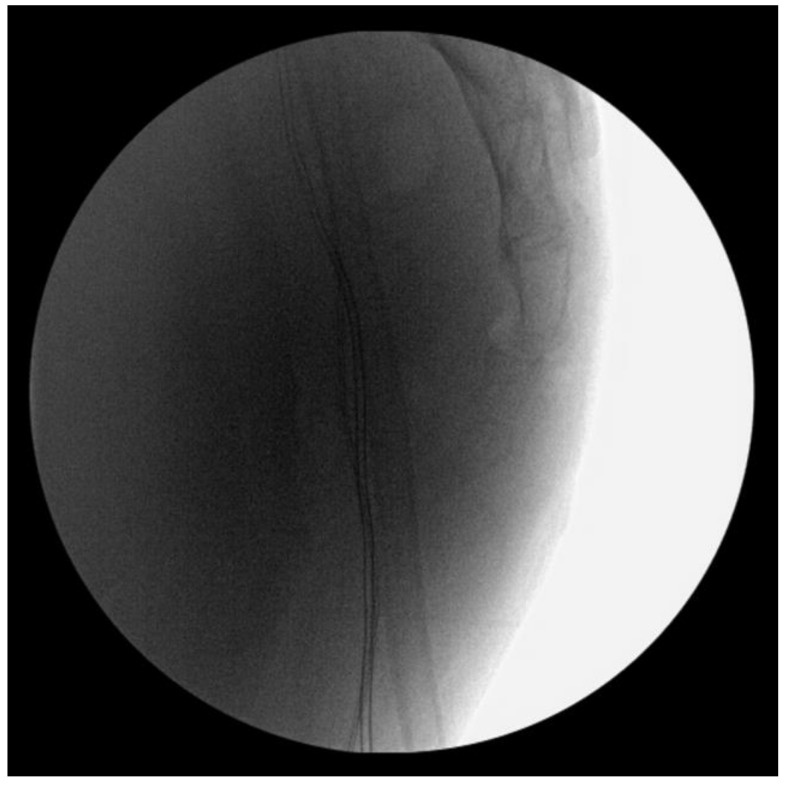
Intraoperative fluoroscopy shows anterior subluxation of the coccyx after removing the freer periosteal elevator.

Intraoperative fluoroscopy confirmed a successful reduction. However, the coccyx tended to be subluxated anteriorly after removing the freer periosteal elevator (Figure 4).

**Figure 5:**
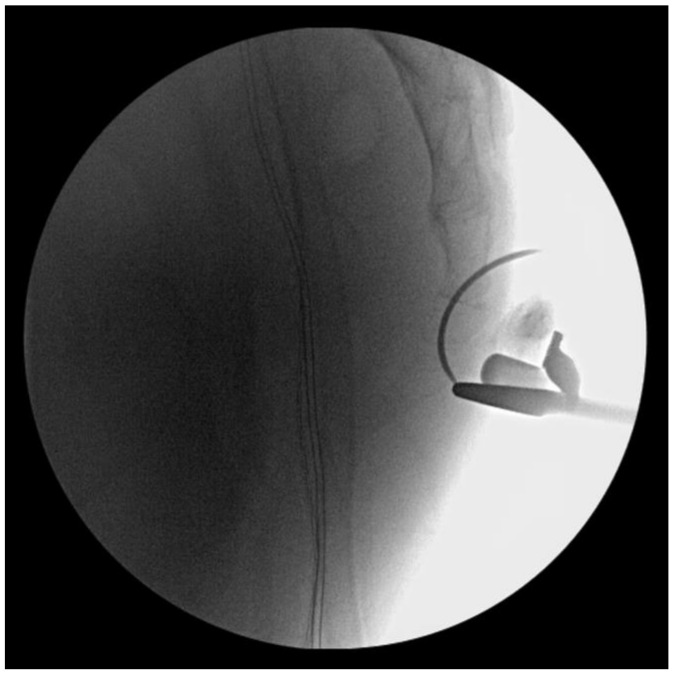
A one-strand trans-osseous vertical suture was performed to maintain the reduction by passing the needle through the skin above and below the incision.

To maintain the reduction, a one-strand trans-osseous vertical suture was performed using 5-Ethibond. To avoid extensive incision, the needle was passed through the skin above and below the incision to ensure penetration into the sacrococcygeal bone (Figure 5).

A mosquito hemostat was used to identify the suture strand in the subcutaneous layer, which was then retrieved through the incision site. Finally, the suture was tied securely. The stability of the joint was confirmed using fluoroscopy.

The patient was allowed to resume full weight-bearing ambulation from the day after surgery. A soft cushion was recommended when sitting or lying down for four weeks after surgery. No restrictions were advised when lying in a supine position. The patient’s symptoms improved significantly after surgery.

**Figure 6:**
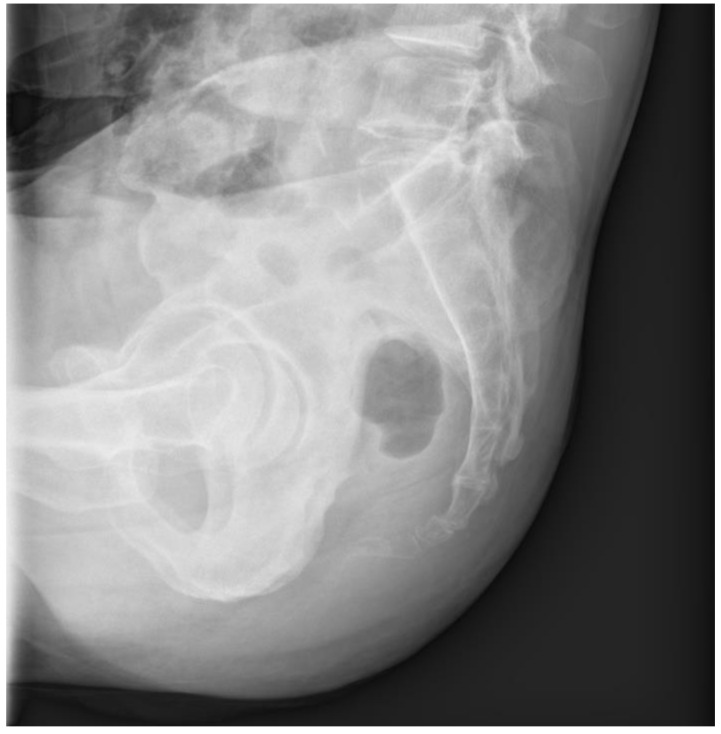
Plain lateral radiography performed six months after the operation. The sacrococcygeal joint maintained good alignment.

At the 6-month follow-up, the sacrococcygeal joint showed good alignment without any additional complications (Figure 6).

## 3. Discussion

Sacrococcygeal dislocation is a rare condition, but it can manifest as a result of traumatic events. Currently, no established treatment with a high level of evidence is available for the management of sacrococcygeal dislocation, and most therapeutic modalities do not guarantee precise realignment of the dislocated joint [5]. In the acute phase, conservative treatments such as the administration of analgesic therapies, the use of soft cushions, and the implementation of physical therapies are utilized to manage pain. Stool softeners may also be prescribed to alleviate pain during bowel movements. Even posterior dislocation has been reported to show good results with conservative treatment [6]. While most patients show pain relief with conservative treatment, residual dislocation can occasionally lead to chronic persistent or recurrent pain. This is called chronic post-traumatic coccygodynia. Coccygodynia, a pathologic condition characterized by pain and discomfort around the lower end of the spine, tends to worsen after extended periods of direct pressure, such as when sitting on hard surfaces. Therefore, in cases where patients do not respond to conservative treatment and to prevent chronic complications, a reduction of the dislocation can be attempted. Additionally, in young women, potential issues related to sexual intercourse and future childbirth should be considered. Currently, the primary methods for reduction include intrarectal manipulation for closed reduction and open reduction via a posterior approach.

Closed reduction is a technique where a gloved finger is inserted through the anus and direct force is applied to the bony step-off of the dislocated sacrococcygeal joint, felt through the posterior rectal mucosa [5]. This method can be used as the initial reduction technique in the acute phase and obviates the need for incision. If the procedure is successful, patients can achieve rapid pain relief and early recovery. However, there are several points to consider. First, cases of failed manual reduction have been reported [3,7]. Raissaki and Williamson [3] reported a case involving a 30-month-old female who had a sacrococcygeal dislocation. They predicted that the child’s anterior dislocation of the coccyx could potentially lead to sexual and labor complications in the future. Given the patient’s young age, they attempted a closed manual reduction under fluoroscopy. However, this attempt was not successful. Second, there is the risk of anterior re-dislocation immediately after the procedure. The tendency to re-dislocate anteriorly is also found during open reduction. In our case, the coccyx tended to slip anteriorly again even after open reduction without additional stabilization. This means that an additional procedure is needed to maintain reduction. Lastly, since a significant amount of force on the rectal mucosa is required to achieve joint reduction, the risk of mucosal damage is increased. Therefore, an initial closed reduction is recommended while preparing for open reduction. Closed reduction must be performed under sufficient anesthesia and muscle relaxation. If closed reduction fails, a transition to open reduction is desirable. Continuing to attempt unreasonable closed reduction may increase the risk of complications.

In 2004, Kim et al. [8] reported a case of minimally invasive open reduction of an acute anteriorly dislocated coccyx. They initially attempted manual reduction through the rectum in the emergency room for anterior sacrococcygeal dislocation that occurred after trauma in a 31-year-old female patient. However, it failed, so open reduction was performed. The open reduction method used in this case was similar to the method reported by Kim et al. To minimize discomfort caused by scarring, a stab incision was made and a freer periosteal elevator was used as a lever, similar to a joystick, to reduce the dislocation of the coccyx under intraoperative fluoroscope. In both cases, anterior slip tended to occur after reduction, requiring an additional procedure for maintenance. Kim et al. achieved stability via the retrograde insertion of a non-threaded Steinmann pin. However, this method required ongoing pin site dressing after surgery, and the patient experienced discomfort due to an inability to lie in the supine position for three weeks after surgery. Furthermore, the Steinmann pin itself carries the risk of irritation or migration. If the pin is cut and buried under the skin to reduce early postoperative discomfort, removal surgery may be needed later. Therefore, we performed one-strand trans-osseous repair using a thick nonabsorbable suture, resulting in the successful maintenance of reduction. Bergkamp and Verhaar [9] performed an open reduction and trans-osseous suture in the same manner as we did. The difference is that they achieved stability via a four-strand tension band suture after reduction. However, a four-strand tension band suture may require a relatively larger incision, and depending on the size of the coccyx, the risk of cortical breakage is high due to the need for several sutures. The one-strand vertical suture performed by the authors was simple and did not require extensive incision, as the suture was passed through the skin and retrieved through the incision site for tying.

The treatment of sacrococcygeal dislocation requires the rapid alleviation of acute pain while minimizing potential complications. For these patients, it is reasonable to try conservative treatment first. However, if pain persists despite conservative treatment or the case is likely to progress to chronic coccygodynia, there is no reason to hesitate with surgery. In this case, the authors were able to achieve pain control and good results without surgery-related complications using the minimally invasive reduction with a one-strand trans-osseous suture technique.

## 4. Conclusions

In conclusion, sacrococcygeal dislocation is usually treated conservatively; however, physicians must consider that residual malposition and instability can lead to chronic complications. For dislocation reduction, try the gentle manual reduction first, and if it fails, a transition to open reduction will be required. The minimally invasive reduction technique with a one-strand trans-osseous suture can be considered as one of the surgical options.

## Data Availability

Not applicable.
